# Prevalence of post-pandemic SARS-CoV-2 in patients with respiratory syndrome in Brazzaville, Republic of the Congo

**DOI:** 10.1186/s12879-025-10541-1

**Published:** 2025-02-14

**Authors:** Jordy Exaucé Demboux Lyelet, Pembe Issamou Mayengue, Félix Koukouikila-Koussounda, Ahmed Jordy Doniama Essialaba, Christ Marty Stéphane Vembe Mahounga, Aldi Fred Mandiangou, Grâce Petula Urielle Fila-Fila, Léadisaelle Hosanna Lenguiya, Novy Charel Bobouaka Bonguili, Eric Elguero, Eric M. Leroy, Pierre Becquart, Fabien Roch Niama

**Affiliations:** 1 Faculté des Sciences et Techniques, Université Marien Ngouabi, Brazzaville, BP69 République du Congo; 2 Laboratoire National de Santé Publique, Brazzaville, BP120 République du Congo; 3 Institut National de Recherche en Sciences de l’Ingénieur, Innovation et Technologie, Cité Scientifique de Brazzaville, route de l’Auberge de Gascogne, Brazzaville, BP181 République du Congo; 4 UMR TransVIHMI, Université de Montpellier-IRD-Inserm, Montpellier, 34394 France; 5 Institut de Recherche pour le Développement, Unité Mixte de Recherche Maladies Infectieuses et Vecteurs, Ecologie, Génétique, Evolution et Contrôle (MIVEGEC), Université Montpellier, Montpellier, 34394 France

**Keywords:** SARS-CoV-2, Post-pandemic, Respiratory syndrome, Republic of the Congo

## Abstract

The first confirmed case of COVID-19 was detected in the Republic of the Congo in March 2020. Several control measures were implemented during the pandemic period. As a result, the number of reported cases decreased significantly, leading to the gradual lifting of barrier measures and the declaration of the end of the outbreak on 14 October 2022. The present study aimed to determine the post-pandemic prevalence of SARS-CoV-2 in the referral hospitals of Talangaï (HRT) and Makélékélé (HRM) in Brazzaville between October 2022 and April 2023. Nasopharyngeal samples collected from patients with respiratory syndrome were analyzed via qRT‒PCR to detect SARS-CoV-2. An overall prevalence of 5% of SARS-CoV-2 infection was found, with monthly fluctuations in cases during the study period, likely reflecting the endemic nature of the infection. The monthly proportion of SARS-CoV-2 infection cases did not correlate with the number of patients with respiratory syndrome-related symptoms. Although the post-pandemic prevalence of SARS-CoV-2 has remained low, laboratory confirmation of COVID-19, which accounts for both clinical suspicion and detection of SARS-CoV-2, using at least one rapid diagnostic test (RDT) is needed to improve case-by-case management in health centers.

## Introduction

Severe acute respiratory syndrome coronavirus 2 (SARS-CoV-2) disease has been prevalent worldwide since December 2019 and has caused a significant loss of life. SARS-CoV-2 primarily affects the respiratory system with a wide range of clinical manifestations, from mild to severe, particularly in elderly and immunocompromised patients [1, 2]. The first alert was an outbreak of pneumonia cases reported in Wuhan, China, on November 17, 2019 [3], after which the disease spread rapidly from country to country.

In December 2023, there were 772,838,745 confirmed cases of COVID-19 worldwide, with 6,988,679 deaths, and more than 8,986,322 confirmed cases were recorded in Africa, with 174,211 deaths [4].

Although serologic evidence of SARS-CoV-2 infection was obtained in the Republic of the Congo in late 2019 [5], the first confirmed case of COVID-19 was detected on 14 March 2020, and the epidemic has progressively evolved in four waves of infection, with the number of corresponding laboratory-confirmed cases and deaths ranging from 7,107 to 108, respectively, at the end of 2020 to 24,775 and 386, respectively, in July 2022 [6, 7]. The Republic of the Congo, like many other African countries, has experienced several variants of SARS-CoV-2, including the Omicron which appeared in December 2021 [8], known for its mild form of infection [9]. Given the significant reduction in cases, presumably due to the impact of vaccination efforts, all barrier measures were removed with case-by-case management instructions implemented in all health districts on 14 October 2022 [10, 11]. COVID-19 was integrated into the standard algorithm for the management of respiratory infections without the need to perform a COVID-19 test. To our knowledge, little is known about post-pandemic SARS-CoV-2 infections in the Republic of the Congo, which may continue to impact globally clinical status of people after having COVID-19 as well as case management of new infection.

We assumed that some COVID-19 cases would be present among respiratory infections recorded in hospitals, which has implications for case management. Therefore, the current study aimed to determine the prevalence of COVID-19 during the post-pandemic period in two referral hospitals in Brazzaville.

## Materials and methods

### Study design

We conducted a prospective cross-sectional surveillance study in hospital settings from March 14, 2022 to April 15, 2023 in Brazzaville, which is now divided into nine arrondissements. Outpatients presenting with a respiratory syndrome as defined by the WHO influenza surveillance protocol were included in two departments (pediatrics and emergency) of the two referral hospitals located in densely populated districts with wide accessibility to the local population. The WHO case definition included: influenza-like illness (ILI), which is the association or succession of an often-hacking cough, with at least one of the following signs: dyspnea, chest pain, wheezing, recent diffuse or focal auscultatory signs, and at least one general sign suggestive of infection (fever, sweats) [12]. The first site, Talangaï Referral Hospital (HRT), is located in the north of Brazzaville and primarily serves patients from the two surrounding arrondissements. The second site, Makélékélé Referral Hospital (HRM), is located in the southern part of the city, and mainly serves patients from three surrounding arrondissements.

### Patients and samples collections

The study population primarily included outpatients of any age from 6 months who presented with respiratory symptoms and gave informed consent (parental consent for children and adolescents). Nasopharyngeal swab samples were collected on site by medical staff during the consultation. A polyester-tipped Dacron swab was inserted into both nostrils until it reached the nasopharynx, then rotated 360° [13]. Swabs were then placed in 3 mL of viral transport medium (VTM). Samples were stored at 4 °C at the collection sites and transported weekly in an electric cooler to the National Public Health Laboratory for molecular analysis. Patients with a pre-existing diagnosis of asthma or sinusitis, and those who were hospitalized and receiving antiretroviral therapy, were excluded from the study. Informed consent was obtained. Each eligible patient was assigned a unique identification number. Sociodemographic and clinical data were collected from participants using a standardized questionnaire to ensure consistent and comprehensive data collection.

### Molecular analysis

#### RNA extraction

Nucleic acids were extracted from nasopharyngeal samples using the NucleoSpin Virus kit (Macherey-Nagel, GmbH & Co. KG, Düren, Germany / Ref: 02/2015, Rev. 02) in accordance with the manufacturer’s instructions [14]. Briefly, the nasopharyngeal samples were previously vortxed for 15 s and 200 µl of the supernatant were used for ARN and DNA purification after the inactivation in the glove box (biosafety box). Nucleic acids were then eluted in 60 µl of RNase/DNase-free elution buffer supplied in the kit, and were either used immediately for viral detection by molecular amplification, or stored at -80 °C for later use.

#### qRT-PCR

Amplification was performed on the QuantStudio 5 [15] using TaqPath Covid-19 CE-IVD RT-PC kits for SARS-CoV-2. The QuantStudio 5 real-time PCR system was used under the following cycling conditions: (1) SARS-CoV-2 by TaqPath: 2 min at 25 °C, 10 min at 53 °C, 2 min at 95 °C, 3 s at 95 °C and 30 s at 60 °C; (2) 21 pathogens by FTD-21: 15 min at 50 °C for reverse transcription, 1 min at 94 °C for denaturation, then 40 cycles for 8 s at 94 °C and 1 min at 60 °C.

### Statistical analyses

Categorical variables were expressed as numbers (%). Fisher exact test and binomial confidence intervals was calculated using SPSS version 23.0 software. A two-sided critical value of alpha = 0.05 was used. A *p*-value of less than 0.05 was considered statistically significant.

## Results

Of the 350 nasopharyngeal samples analyzed, 238 were obtained from children aged 6 months to 17 years. A total of 18 samples were positive for SARS-CoV-2 (18/350; 5%). The sex ratio among these patients was 1, and the median age was 2 years (range, 6 months to 59 years). At the HRT, 4 samples were positive, 3 of which were from pediatric patients, whereas at the HRM, 14 samples were positive, 9 of which were from pediatric patients (Table 1).

**Table 1:** Demographic characteristics and prevalence of SARS-CoV-2 infections in patients

Characteristics	Total	Infected	
	*N* (%) = 350	*n* (%) = 18	95% IC
Sex			
Male	171(48.9)	9(50)	0.6–1.5
Female	179(51.1)	9(50)	0.6–1.6
Age group (years)			
[0–4]	194(55.4)	10(55.5)	0.3–2.5
[5–15]	37(10.6)	3(16.7)	0.1–0.2
[16–87]	119(34)	5(27.8)	0.4–3.9
Study sites			
HRT	153(43.7)	4(22.2)	0.1–1
HRM	197(56.2)	14(77.8)	0.9–8.8
Symptoms			
Fever	173(49.4)	11(61.1)	0.7–7.8
Cough	281(80.2)	14(77.8)	0.2–2.4
Sore throat	100(28.5)	6(33.3)	0.1–2.2
Breathing difficulty	160(45.7)	10(55.5)	0.3–2.6
Nasal discharge	263(75.1)	11(61.1)	0.2–2.1

**Fig. 1:**
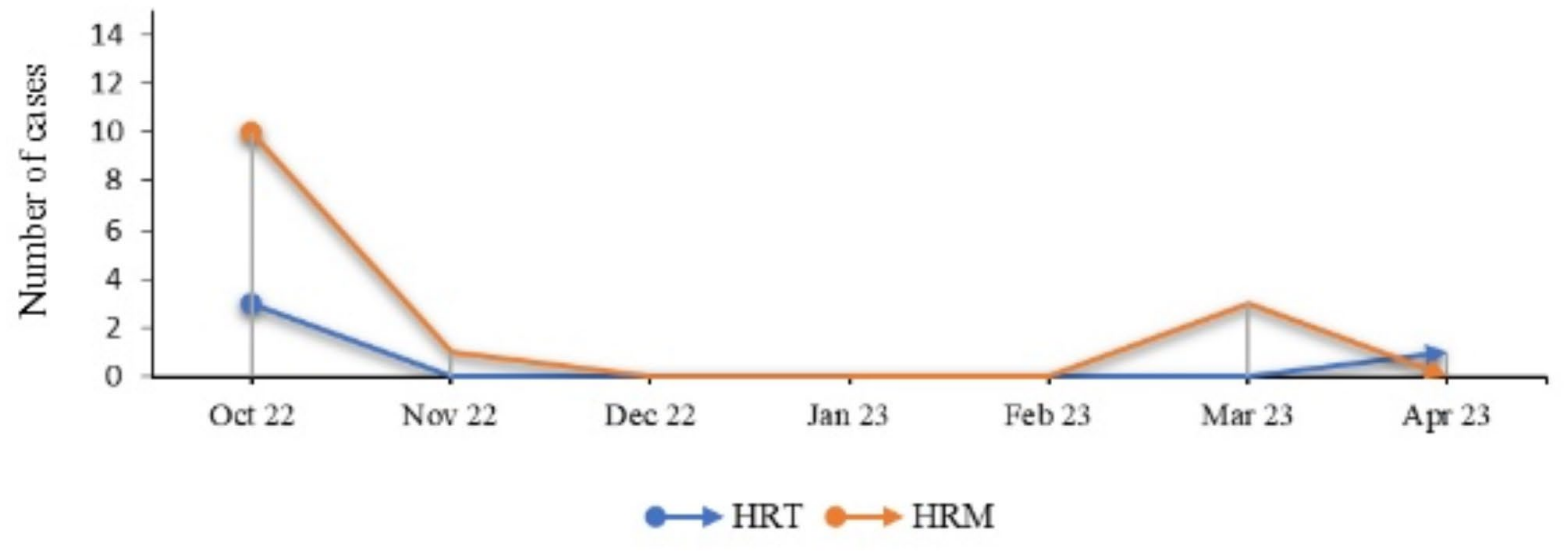
Variation in SARS-CoV-2 infection cases over the study period

Regarding the distribution of cases over the study period, the number of cases was significantly different between the two sites (*p* = 0.04), with a greater number of cases recorded at HRM in October. The number of cases decreased dramatically from October to November 2022, regardless of the site (Fig. 1).

From December 2022 to February 2023, no positive cases were detected at either site. However, three cases were detected in March 2023 at HRM, and one was detected in April 2023 at HRT.

**Fig. 2:**
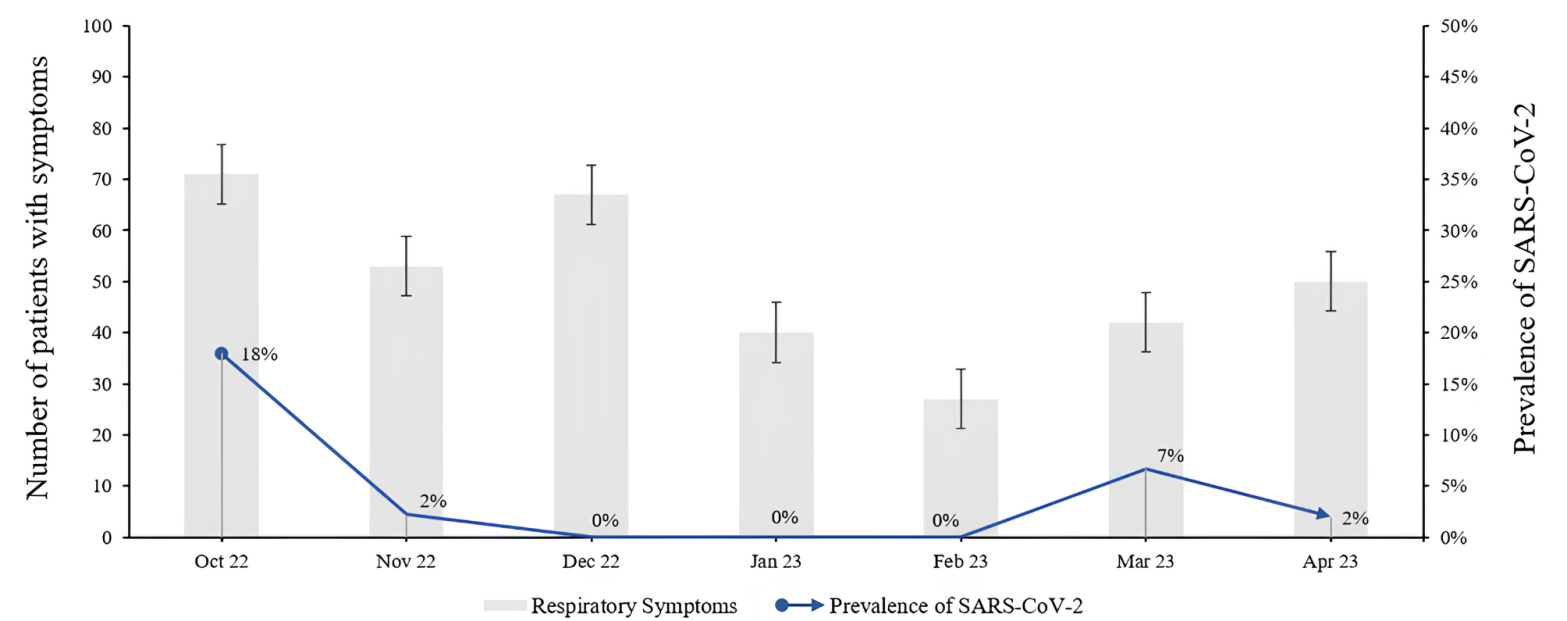
Distribution of SARS-CoV-2 infection cases according to symptoms over the study period

The monthly variation in patients with respiratory syndrome-related symptoms was not normally distributed among the SARS-CoV-2 infection patients (Fig. 2).

**Fig. 3:**
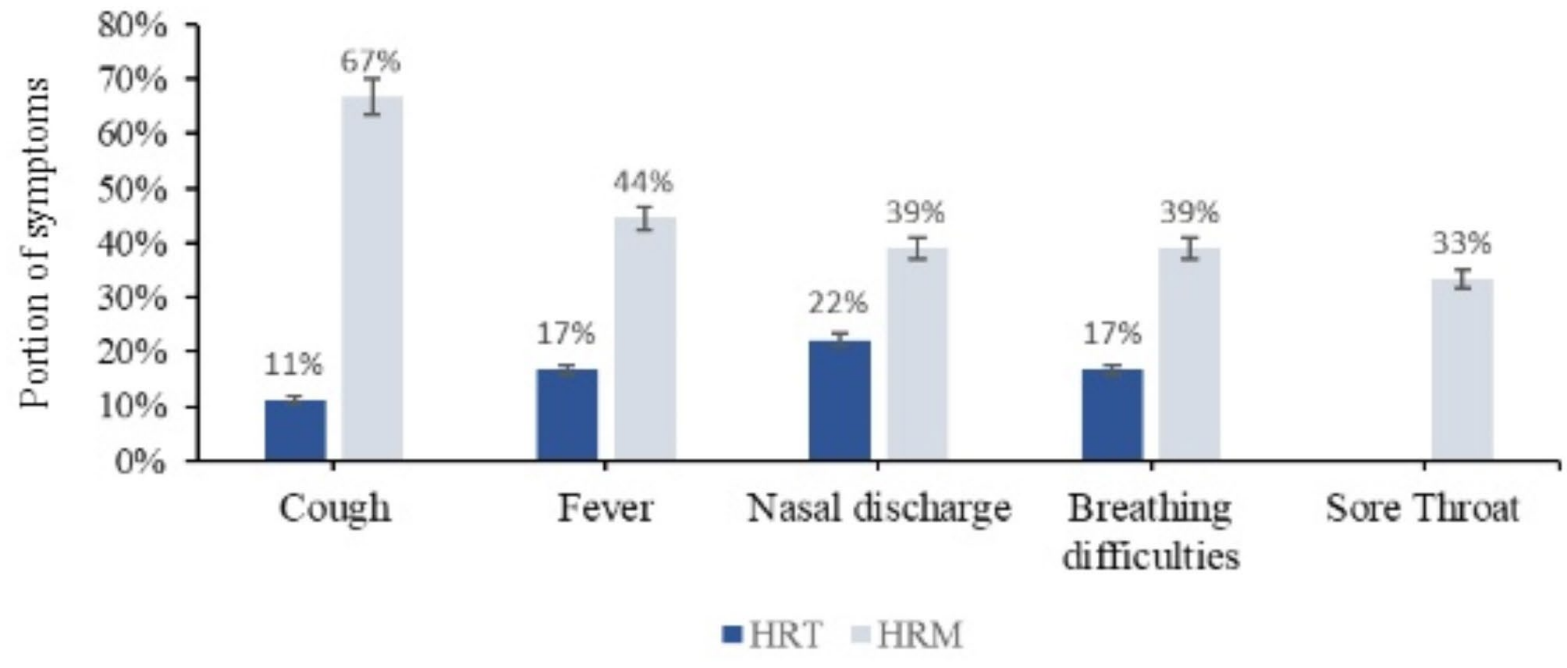
Distribution of symptoms in patients infected with SARS-CoV-2 by location

The most common symptoms found in SARS-CoV-2-infected patients at both sites were cough (78%), fever and nasal discharge (61%), and breathing difficulties (56%), with HRM having the highest proportion of these symptoms (Fig. 3).

However, none of these symptoms showed a statistically significant association (*P* > 0.05) with SARS-CoV-2 infection.

## Discussion

The prevalence of SARS-CoV-2 case was 5%, with a dramatic decrease following the removal of all barrier measures, in line with the government’s policy. However, because new cases were detected four months after the removal of these measures, a diagnosis of COVID-19 considering both clinical suspicion and detection of SARS-CoV-2 using at least one rapid diagnostic test (TDR) is needed for better case-by-case management in all health districts.

The post-pandemic incidence of SARS-CoV-2 infection (5%) found in the present study was lower than that found during the same period in Bukavu in the Democratic Republic of the Congo (DRC) [16] and the Center-West region of Brazil [17], which had 14% and 13%, respectively, despite the similarity of the study populations. In Bukavu, the study population consisted of people with influenza-like illness recruited from different health facilities [16], while in Brazil, the study population consisted of pediatric patients with severe acute respiratory syndrome (SARS) [17]. This difference in the post-endemic prevalence of SARS-CoV-2 infections may be related to epidemiological parameters of the disease, particularly the infection rate and the prevalence of circulating variants during the study period, which are not the same in these different countries.

Although pediatric services were most common at both sites, SARS-CoV-2 infection was more common in patients at HRM than in those at HRT throughout the study period. This difference can be explained by several factors, including differences in sample sizes and vaccination statuses. Indeed, we found that more patients recruited at the time of HRT were vaccinated against COVID-19 than were those recruited at the time of HRM. The high percentage of positive samples in pediatric patients combined with the high number of samples collected could be explained by the fact possibly because Congolese adults less frequently seek medical attention for respiratory syndromes.

Fluctuations in the proportion of SARS-CoV-2 infections were observed during the study period, this fluctuation could relate to seasonal infection patterns, reflecting the endemic characteristics of the virus. However, the consistent presence of symptoms throughout the study period suggested that other respiratory viruses such as influenza or syncytial respiratory virus could also be responsible for the observed symptoms. Indeed, SARS-CoV-2 infection has clinical manifestations similar to those of other respiratory viruses. Due to the lacking of published epidemiolocal data on respiratory viruses in the Republic of the Congo and since different symptoms did not correlate with the incidence of SARS-CoV-2 infection, a study aimed at determining the majority of the respiratory viruses circulating in Brazzaville and their coinfection is needed for better case management.

### Limitation and strengths

(1) the 7-month duration of this study is an important limitation. Thus, longer studies are needed to determine the real impact of lifting barrier measures against SARS-CoV-2 in Brazzaville. (2) the second limitation is the lack of data on asymptomatic cases of SARS-CoV-2 in households to consider all types of infection.

However, this study is a significant step forward on the improvement of the case management of patients with acute respiratory infections.

## Data Availability

The datasets used and/or analysed during the current study are available from the corresponding author on request.

## Abbreviations

SARS-CoV-2: Severe acute respiratory syndrome-associated coronavirus 2
COVID-19: Coronavirus disease 2019
WHO: World health organization
HRT: Talangaï referral hospital
HRM: Makélékélé referral hospital
TDR: Rapid diagnostic test
qRT‒PCR: Quantitative real-time polymerase chain reaction
CERSSA: Comité d’Ethique de la recherche en sciences de la Santé

## References

[1] Desai AD, Lavelle M, Boursiquot BC, Wan EY. Long-term complications of COVID-19. Am J Physiology-Cell Physiol. 2022;322(1):C1–11. 10.1152/ajpcell.00375.2021 PMC8721906 34817268

[2] WHO Coronavirus (COVID-19) Dashboard with Vaccination Data. https://covid19.who.int/. Accessed 8 Mar 2022.

[3] Josephine, M: Coronavirus: China’s first confirmed Covid-19 case traced back to November 17, South China Morning Post, 13 mars 2020. https://www.scmp.com/news/china/society/article/3074991/coronavirus-chinas-first-confirmed-covid-19-case-traced-back?module=perpetual_scroll_0&pgtype=article. Accessed 6 Jan 2021.

[4] COVID-19. WHO African region. https://who.maps.arcgis.com/apps/dashboards/0c9b3a8b68d0437a8cf28581e9c063a9. Accessed 3 May 2023.

[5] Bonguili NCB, Fritz M, Lenguiya LH, Mayengue PI, Koukouikila-Koussounda F, Dossou-Yovo LR, Niama FR. Early circulation of SARS-CoV-2, Congo, 2020. Emerg Infect Dis. 2022;28(4):878. 35180374 10.3201/eid2804.212476 PMC8962888

[6] WHO. Response to the COVID-19 epidemic in Congo. Situation reports. Regional Office for Africa (who.int), SITREP N°244 du 31 mars 2020. https://www.afro.who.int/fr/countries/congo/publication/riposte-lepidemie-de-covid-19-au-congo-rapports-de-situation. Accessed 18 jun 2024.

[7] WHO. Response to the COVID-19 epidemic in Congo. Situation reports Regional Office for Africa (who.int), SITREP N°224 du 25 Juillet 2022. https://www.afro.who.int/fr/countries/congo/publication/riposte-lepidemie-de-covid-19-au-congo-rapports-de-situation. Accessed 18 jun 2024.

[8] WHO EMRO. Variant Omicron: ce qu’il faut savoir COVID-19, Thèmes de santé. https://www.emro.who.int/fr/health-topics/corona-virus/omicron-voc-questions-and-answers.html#:~:text=Le%20variant%20Omicron%20ne%20devrait,infection%20par%20le%20variant%20Omicron. Accessed 23 jun 2024.

[9] WHO. Le travail de l'Organisation Mondiale de la Santé en République du Congo. Rapport annuel 2021. https://www.afro.who.int/sites/default/files/2023-06/Rapport%20annuel%202021%20.pdf. Accessed 28 jul 2024

[10] WHO. Response to the COVID-19 epidemic in Congo. Situation reports Regional Office for Africa (who.int), communiqué de la coordination nationale de gestion de la pandémie de coronavirus covid 19. https://www.afro.who.int/fr/countries/congo/publication/riposte-lepidemie-de-covid-19-au-congo-rapports-de-situation. Accessed 18 jun 2024.

[11] Gouvernement Congolais. Communiqué de la. coordination nationale de gestion de la pandémie de coronavirus covid-19 suite à sa réunion du vendredi 14 octobre 2022. https://gouvernement.cg/communique-de-la-coordination-nationale-de-gestion-de-la-pandemie-de-coronavirus-covid-19-suite-a-sa-reunion-du-vendredi-14-octobre-2022/. Accessed 30 jun 2024.

[12] WHO. surveillance case definitions for SARI. and ILI. https://www.who.int/teams/global-influenza-programme/surveillance-and-monitoring/case-definitions-for-ili-and-sari. Accessed 30 jun 2024

[13] Janahi I, Abdulkayoum A, Almeshwesh F, Alkuwari M, Hammadi A, A., Alameri M. Viral aetiology of bronchiolitis in hospitalised children in Qatar. BMC Infect Dis. 2017;17:1–11. 28193180 10.1186/s12879-017-2225-z PMC5307797

[14] NucleoSpin Virus. RNA and DNA purification user Manual_Rev_02.Pdf (takarabio.com).

[15] QuantStudio™ 5 Real-Time PCR System for Human Identification, 96-well, 0.2 mL, desktop. https://www.thermofisher.com/order/catalog/product/A34322. Accessed 16 jul 2024.

[16] Ntagereka PB, Basengere RA, Baharanyi TC, Kashosi TM, Buhendwa JC, Bisimwa PB, et al. Molecular evidence of coinfection with acute respiratory viruses and high prevalence of SARS-CoV-2 among patients presenting flu-like illness in Bukavu city, Democratic Republic of Congo. Can J Infect Dis Med Microbiol. 2022. 10.1155/2022/1553266. 10.1155/2022/1553266 PMC8994514 35411212

[17] Malveste Ito CR, Moreira ALE, Silva PAND, Santos MO, Santos APD, Rézio GS, et al. Viral coinfection of children hospitalized with severe acute respiratory infections during COVID-19 pandemic. Biomedicines. 2023;11(5):1402. 10.3390/biomedicines11051402. 10.3390/biomedicines11051402 PMC10216660 37239073

